# Association between iron metabolism and cognitive impairment in older non-alcoholic fatty liver disease individuals: A cross-sectional study in patients from a Chinese center

**DOI:** 10.1097/MD.0000000000018189

**Published:** 2019-11-27

**Authors:** Jing Xu, Weihao Sun, Li Yang

**Affiliations:** a Department of Geriatric Gastroenterology, The First Affifiliated Hospital of Nanjing Medical University; b Department of Geriatrics, Nanjing Drum Tower Hospital, The Affiliated Hospital of Nanjing University Medical School, Nanjing, China.

**Keywords:** aged, mild cognitive impairment, non-alcoholic fatty liver disease, soluble transferrin receptor

## Abstract

Sparse is the research on the relationship between iron metabolism and mild cognitive impairment (MCI) in adults aged over 60 years with non-alcoholic fatty liver disease (NAFLD). The soluble transferrin receptor (sTfR), serum iron (SI), serum ferritin (SF), transferrin (TRF) and hemoglobin (HB) are indicators of iron metabolism.

This study examined whether iron metabolism is associated with cognitive impairment in older individuals.

A cross-sectional study was held in patients from a Chinese center. Individuals with NAFLD aged over 60 years were included if they did not have excessive alcohol intake and were free of stroke or dementia. Their cognitive function was assessed by the same neurologist. 3.0T H proton magnetic resonance spectroscopy (^1^H-MRS) was performed to evaluate the hippocampus of the participants without contraindication. *t* test and Chi-square test were used to analyze the data. Binary logistic regression was used for correlation analysis.

Fifty four (54%) of participants were diagnosed with MCI by the psychiatrist. MCI was significantly associated with higher sTfR after adjustment of all the covariates (*OR* = 2.565, *95%CI*: 1.334∼4.934; *P* = .005). No statistically significant associations were observed between MCI and age or blood glucose or choline (Cho) /creatine (Cr) of theright hippocampus head.

Increased age and low levels of sTfR and HB were associated with MCI in NAFLD individuals aged over 60 years.

## 1 Introduction

Non-alcoholic fatty liver disease (NAFLD), an acquired metabolic stress liver disease, refers to a clinicopathological syndrome characterized by diffuse bullous steatosis of hepatocytes, after exclusion of alcohol and other definite liver-damaging factors. The pathogenesis of NAFLD is complex. As the extension of visceral adipose tissue, heterotopic liver induces lipid-forming changes, insulin resistance, oxidative stress/lipid peroxidation and inflammatory damage and exacerbates peripheral insulin resistance and systemic low-grade inflammation. Therefore, NAFLD not only disrupts liver function, but also triggers complex extrahepatic metabolic processes.

A study has found that NAFLD is an independent risk factor for coronary heart disease and heart failure.^[1]^ Compared with healthy adults, NAFLD patients without cardiovascular risk factors (such as hypertension and diabetes) still have left ventricular hypertrophy and left ventricular diastolic function changes.^[2]^ NAFLD also affects the brain. Quantitative analysis of the whole brain, hippocampus and white matter with magnetic resonance imaging(MRI) reveals reduced brain volume, decreased serial digital learning test level,^[3]^ visual spatial impairment and executive function damage in NAFLD patients.^[4]^ However, all the previous clinical studies put focus on young and middle-aged NAFLD patients, never on the elderly aged over 60 years.

Our previous study found that NAFLD patients of over 60 years had decreased cognitive function and increased soluble transferrin receptor (sTfR) compared with non-NAFLD people of the same age.^[5]^ According to the Guidelines for the Diagnosis and Treatment of Dementia and Cognitive Impairment in China in 2018, metabolic factors, including blood pressure, blood lipid, blood sugar and obesity, may increase MCI risk in older people.^[6]^ However, it is not known whether damaged cognitive function is associated with iron metabolism in older NAFLD patients. This is despite evidence of abnormal ferrugination in the liver of NAFLD patients^[7]^ and in the hippocampus of Alzheimer disease (AD) patients.^[8]^ The biopsy of the liver and the hippocampus, the gold standard for abnormal iron deposition, is rarely used for clinical assessments because of its invasiveness. In the present study, we used noninvasive testing to detect the iron metabolism in NAFLD patients, aiming to tease out the association between iron metabolism and cognitive function in older NAFLD patients.

## 2 Methods

### 2.1 Participants

This cross-sectional study was conducted at Nanjing Drum Tower Hospital (Nanjing, China). The study recruited adults aged over 60 years, who were diagnosed with NAFLD in the Geriatrics Department of Nanjing Drum Tower Hospital between December 2014 and June 2016. Excluded were those with alcoholic liver disease, viral hepatitis, drug-induced liver disease and other specific diseases that can lead to fatty liver; serious heart, liver, renal insufficiency and cerebrovascular diseases; malignant tumors and other end-stage diseases; stroke, dementia orother mental disorders. Because the older patients often suffer from a variety of diseases (such as severe cognitive impairment, stroke, anemia, malnutrition, heart failure and so on) that affect cognitive function or iron metabolism assessment, we only recruited 100 eligible volunteers in nearly two years. All participants gave their written informed consent. The protocol was approved by the Ethical Review Board of Nanjing Drum Tower Hospital.

### 2.2 Measurements

The medical reports of all the patients were assessed to confirm the presence of NAFLD. The age, gender, height, weight, body mass index (BMI), waist hip ratio, blood pressure, duration of NAFLD, glycosylated hemoglobin (HbA1c), hemoglobin (HB), iron metabolism indices, and lipid profile were recorded. The medical history of each patient was comprehensively reviewed, including mini mental state examination (MMSE), Montrealcognitive assessment (MoCA), soluble transferrin receptor (sTfR), serum iron (SI), serum ferritin (SF) and transferrin (TRF). The diagnosis of MCI was made by neurologists.

MMSE and MoCA are globally used instruments to assess cognitive functions, like orientation, attention, executive functioning, and memory. A clinical neuropsychologist (research assistant or psychologist/psychiatrist) was trained to assess the cognitive function of participants.

3.0T H proton magnetic resonance spectroscopy (^1^H-MRS) was performed in the hippocampus of 49 patients without contraindication. The data was processed by magnetic resonance postprocessing workstation. The hippocampus was divided into three pans (head, body and tail) and the ratios of N-acetylaspartate (NAA) /creatine (Cr). myoinositol (MI) /Cr. MI/NAA and choline (Cho) /Cr were calculated separately.^[9]^

### 2.3 Statistical analysis

According to the general MCI criteria,^[5]^ 100 patients were assigned to the MCI (n = 54) or Non-MCI group (n = 46). We analyzed entirety characteristics of the 2 groups, including gender, smoking, income, residence, education, family and so on. The obtained data were analyzed using SPSS 19.0. Descriptive and inferential statistics were calculated to present the data. Chi-square test and independent sample *t* test were used to test the mean between the 2 groups. Multinomial logistic regression was used to determine the contribution of NAFLD to cognitive impairment. Results were reported as relative risk ratios and 95% confidence intervals. Two-tailed *P* value < .05 was considered statistically significant.

## 3 Results

The MCI group had significantly higher mean age and Cho/Cr of right hippocampus head (84.7 ± 7.1 years, 1.27 ± 0.25) than the non-MCI group (78.0 ± 9.1 years, 0.94 ± 0.31; Tables 1 and 2). No significant differences were found in hippocampal height, choroidal fissure, temporal lobe trunk and lateral fissure cistern width between the two groups. MCI group showed sTfR and HB (5.5 ± 2.6, 127.3 ± 17.5) lower than those in non-MCI group (10.2 ± 2.5, 134.5 ± 13.8; Table 3), while SI, SF and TRF presented no significant differences.

**Table 1:**
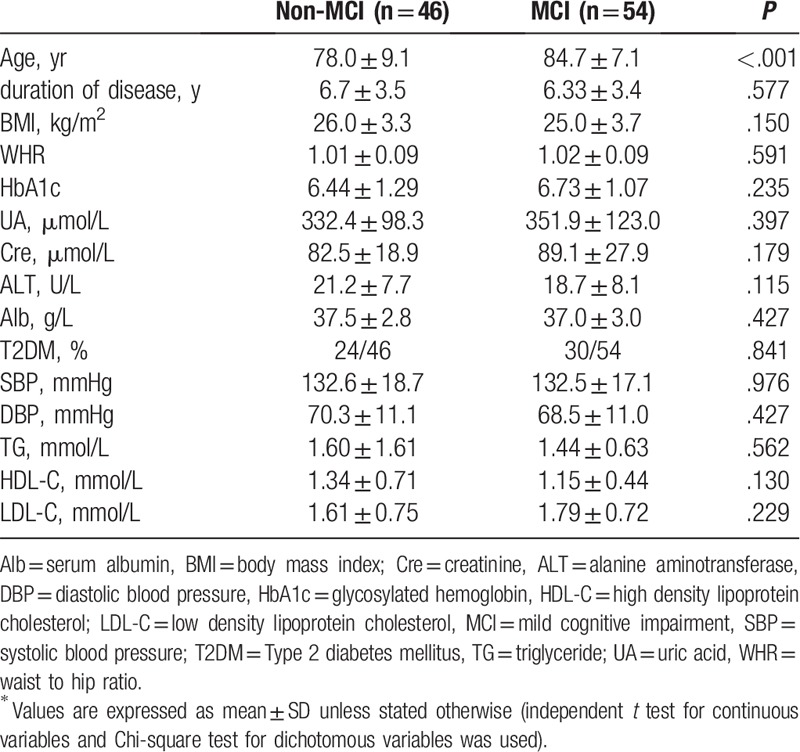
Baseline characteristics of the patients^∗^.

**Table 2:**
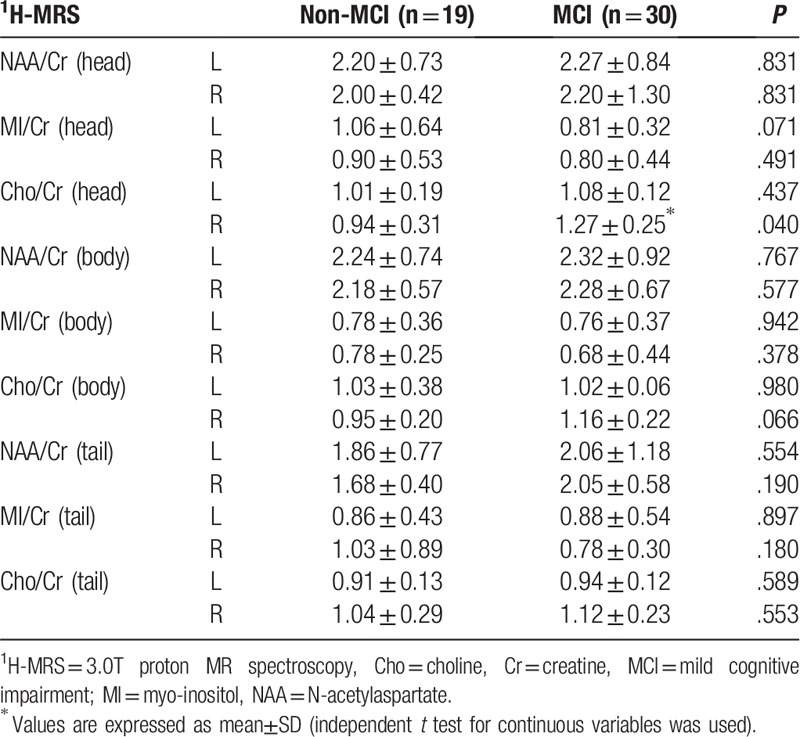
Biochemical indexes in hippocampu of the Patients^∗^.

**Table 3:**
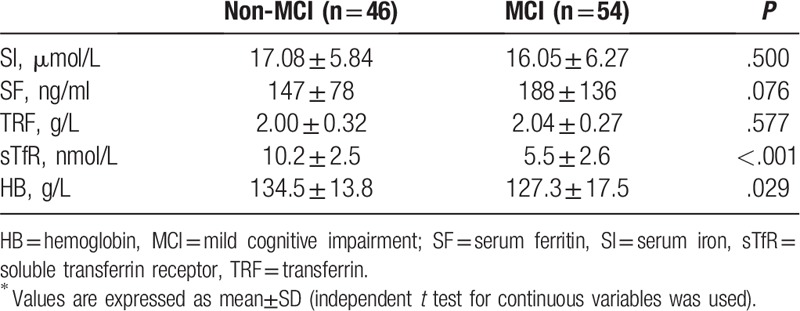
Iron metabolism indicators of the patients^∗^.

Factors (such as age, right hippocampal head Cho/Cr, sTfR and HB) were taken as independent variables. The presence of cognitive impairment was taken as a dependent variable. Multivariate logistic regression model was constructed. After confounding factors were excluded, we found that sTfR was an independent factor of NAFLD combined with MCI (OR = 2.565: 95%CI: 1.334∼4.934: *P* = .005).

## 4 Discussion

In our previous report, older NAFLD individuals were prone to cognitive dysfunction compared with those of the same age without NAFLD.^[5]^ In the present study, increased age, sTfR and Cho /Cr of right hippocampus head were correlated with MCI. Binary logistic regression analysis showed negative correlation between sTfR and cognitive function.

Iron molecules can deposit in microglia and astrocytes of the endothelium, cerebellum, substantia nigra, hippocampus, which may lead to cognitive impairment in severe cases. An autopsy of 11 patients with cognitive impairment by the Institute of Psychiatry of University of London showed that the iron content in the patients’ superior temporal gyrus was 15 to 20 times higher than that in the control group.^[8]^ This pathological change was closely related to gender and iron metabolism genes.^[7]^ Our previous study also found that the abnormal expression of iron metabolism genes occurred before the decline of learning and memory ability in animals,^[10]^ suggesting that the molecular biological changes in the brain tissues may occur earlier than behavioral abnormalities. In addition to iron deposition in neurons caused by abnormal expression of iron metabolism genes, peripheral iron load is also a cause of cognitive impairment. Ferri ions in blood circulation pass through blood-cerebrospinal fluid barrier by transferrin/transferrin receptor and endosome. Most ferri ions traveling through the blood-cerebrospinal fluid barrier bind to transferrin synthesized and secreted by astrocytesand hcpec, and then are absorbed by transferrin/transferrin receptor pathway on neuronal surface. Therefore, iron overload in the peripheral circulation can lead to iron deposition in the central nervous system.

Liver, the major organ that stores excess iron in human body, has become the primary target organ of iron toxicity damage caused by either primary or secondary iron overload. In 1994, iron overload was first reported in NAFLD patients. The subsequent pathological evidence showed that 71% of patients with steatohepatitis and 50% of patients with simple steatosis were positive for iron staining.^[7]^ In the present research, we found that the serum sTfR level was significantly decreased. The SF increased without statistically significant difference. Although sTfR is a sensitive marker of iron deficiency diseases,^[11]^ it is an independent predictor and may not be related to iron metabolism. For example, the subjects with severe pre-existing coronary artery disease had increased levels of sTfR. Coronary artery disease patients with acute coronary syndromes showed increased levels of serum ferritin.^[12]^ Therefore, whether cognitive function in older patients with NAFLD is related to abnormal iron metabolism or only to sTfR needs further study. To confirm the correlation between MCI and abnormal iron metabolism, polymorphism of sTfR upstream gene and protein expression will be detected in future studies.

The hippocampus, a part of the limbic system of the brain, is responsible for the storage, conversion and orientation of long-term memory. MRI has confirmed that in early AD patients, the brain tissue changes, mainly confined to the hippocampus, are manifested by hippocampal volume atrophy.^[13]^ Current studies on hippocampal structural changes in patients with MCI are inconsistent, partly because early brain structural atrophy in some areas can be compensated by different degrees of glial cell proliferation. Therefore, it is difficult to evaluate the brain tissue volume and diagnose early-stage MCI by structural MRI alone. The development of functional magnetic resonance technology allows the application of ^1^H-MRS, a new non-invasive method to measure metabolites in the central nervous system.^[14]^^1^H-MRS detects tiny abnormal biochemical metabolism that is undetectable by traditional imaging techniques in the living tissues.^1^H-MRS technology can reveal peak curves of some specific nuclei in the region of interest and the signals containing corresponding nuclear compounds, as well as the chemical shift action, and finally the metabolite curves of region of interest is produced.

^1^H-MRS can detect neurobiochemical metabolites such as NAA, MI, Cho and Cr. NAA is a biochemical marker of neurons, most abundant in neurons, dendrites and axons. When neuron apoptosis or energy metabolism disorder occurs, the NAA concentration decreases.^[15]^ MI is involved in the formation of osmotic pressure and surfactant. The increase of MI concentration suggests the proliferation of glial cells.^[16]^ Cho, which mainly exists in neurons and glial cells, maintains the cell membrane structure and participates in the formation of myelin sheath. The increase of Cho suggests the accelerated catabolism of nerve cell membrane, a compensatory manifestation of nerve cell damage.^[17]^ Cr is a nitrogen-containing organic acid that acts as an energy buffer in brain cells. Its absolute concentration is not affected by brain metabolism, and its distribution is uniform and relatively constant, so it is often used as an indicator to measure the absolute concentration of other metabolites.^[18]^

In the present study, MRI of hippocampus was performed in 49 patients without any contraindication (like presence of cardiac pacemakers and metal implants). There was no significant difference in hippocampal height, choroidal fissure, temporal lobe trunk and lateral fissure cistern width between the MCI and non-MCI groups. Neurometabolism in hippocampal head to tail^[19]^ showed no significant correlation between increased Cho/Cr and the change of cognitive function despite the elevated Cho/Cr in the right hippocampal head of MCI patients. This may be explained by the different fiber connections between the hippocampal head and the body. Compared with the head, both the body and the tail behind the dentate gyrus of the hippocampus are more involved in visual spatial memory coding.^[20,21]^ The small sample size may also lead to negative results. Although this research was based on a comparatively small size, our well-designed analytic methods and parameters still made the results reliable.

In conclusion, increased age and low levels of sTfR and HB were associated with NAFLD and MCI in older individuals.

## Acknowledgments

The author is very thankful to all the associated personnel in any reference that contributed in/for the purpose of this research.

## Author contributions

**Conceptualization:** Jing Xu.

**Data curation:** Li Yang.

**Funding acquisition:** Jing Xu.

**Investigation:** Jing Xu.

**Methodology:** Jing Xu, Li Yang.

**Supervision:** Sun Weihao.

**Writing – original draft:** Jing Xu.

**Writing – review & editing:** Sun Weihao.
